# Influence of heat stress on intestinal integrity and the caecal microbiota during *Enterococcus cecorum* infection in broilers

**DOI:** 10.1186/s13567-022-01132-y

**Published:** 2022-12-16

**Authors:** Jana Schreier, Ivan Rychlik, Daniela Karasova, Magdalena Crhanova, Gerhard Breves, Silke Rautenschlein, Arne Jung

**Affiliations:** 1 Clinic for Poultry, University of Veterinary Medicine Hannover, Foundation, Buenteweg 17, 30559 Hannover, Germany; 2 Veterinary Research Institute, Hudcova 296/70, 62100 Brno, Czech Republic; 3 Institute for Physiology and Cell Biology, University of Veterinary Medicine Hannover, Foundation, Bischofsholer Damm 15, 30173 Hannover, Germany

**Keywords:** *Enterococcus cecorum*, heat stress, intestinal integrity, caecal microbiota, broiler

## Abstract

*Enterococcus cecorum* (EC) is one of the most relevant bacterial pathogens in modern broiler chicken production from an economic and animal welfare perspective. Although EC pathogenesis is generally well described, predisposing factors are still unknown. This study aimed to understand the effect of heat stress on the caecal microbiota, intestinal integrity, and EC pathogenesis. A total of 373 1-day-old commercial broiler chicks were randomly assigned to four groups: (1) noninoculated, thermoneutral conditions (TN); (2) noninoculated, heat stress conditions (HS); (3) EC-inoculated, thermoneutral conditions (TN + EC); and (4) EC-inoculated, heat stress conditions (HS + EC). Birds were monitored daily for clinical signs. Necropsy of 20 broilers per group was performed at 7, 14, 21, and 42 days post-hatch (dph). A trend towards enhanced and more pronounced clinical disease was observed in the EC-inoculated, heat-stressed group. EC detection rates in extraintestinal tissues via culture were higher in the HS + EC group (~19%) than in the TN + EC group (~11%). Significantly more birds were colonized by EC at 7 dph in the HS + EC group (100%) than in the TN + EC group (65%, *p* < 0.05). The caecal microbiota in the two EC-inoculated groups was significantly more diverse than that in the TN group (*p* < 0.05) at 14 dph, which may indicate an effect of EC infection. An influence of heat stress on mRNA expression of tight junction proteins in the caecum was detected at 7 dph, where all six investigated tight junction proteins were expressed at significantly lower levels in the heat stressed groups compared to the thermoneutral groups. These observations suggest that heat stress may predispose broilers to EC-associated disease and increase the severity thereof. Furthermore, heat stress may impair intestinal integrity and promote EC translocation.

**Supplementary Information:**

The online version contains supplementary material available at 10.1186/s13567-022-01132-y.

## Introduction

*Enterococcus cecorum* (EC) is one of the most common bacterial pathogens in meat-type chickens worldwide [1–7]. EC-associated disease is characterized by progressive lameness or symmetrical paresis of the legs in the second half of the production cycle [1, 3]. Prior to this chronic stage, affected birds can be either asymptomatic or show nonspecific symptoms of septicemia [8]. Antibiotic therapy is only promising if it is started in time during the septic phase. Severely affected birds need to be culled in both stages of the disease [1, 3, 5, 8]. Despite the resulting economic losses and animal welfare issues, there is still a lack of knowledge regarding the pathogenesis of EC-associated disease, such as virulence factors of EC and predisposing factors for the disease [9, 10].

Pathogenic EC strains colonize the intestines of broiler chickens after oral infection within the first week of life [11]. In the second to third week of life, EC translocates to the bloodstream. Affected birds suffer from septicemia, and EC can be detected in several extraintestinal organs, including the heart, liver, or spleen [8, 11]. Subsequently, EC reaches osseous predilection sites at the free thoracic vertebra and the femoral heads via the bloodstream, where it may colonize *osteochondrosis dissecans* (OCD) lesions. These osteochondrotic lesions usually develop due to strong mechanical stress at the weight-bearing vertebral and coxofemoral articulations. The resulting formation of necrotic abscesses is the cause of lameness and paresis [11, 12]. Thus far, it is unknown which virulence factors enable EC to translocate from the gut to other tissues and how the caecal microbiota composition and intestinal barrier function might affect this translocation process and vice versa [9]. However, several predisposing factors in EC pathogenesis have been discussed. A recent study indicated that inadequate ventilation of the barn during the first days of life, shortened dark periods, and inadequate cleaning and disinfection between cycles increase the risk of EC-associated disease occurrence [13]. The impact of housing temperature on EC infection has not been studied. Nonetheless, it has been shown that heat stress might be a predisposing factor for intestinal colonization by pathogenic bacteria such as *Clostridium perfringens* [14], *Salmonella* Enteritidis [15], and *Escherichia coli* [16]. Heat stress is an environmental factor that can exert a decisive impact on chicken health in general and on the intestinal microbiota and intestinal integrity in particular [17].

The chicken intestinal microbiota interacts predominately with dietary compounds, the intestinal mucosa, and the immune system of the host. Consequently, it is of vital importance for chicken health [18–20]. The composition of the chicken caecal microbiota varies depending on diet, age, and housing [21–23]. Seasonal and environmental factors can substantially influence the development of the microbial community in chickens [24, 25]. Marked changes in the gut microbial composition have been found in heat-stressed broilers with decreased abundances of beneficial bacteria (e.g., *Lactobacillus* spp. and *Bifidobacterium* spp.) and increased abundances of coliforms and *Clostridium* spp. [26]. It has been speculated that heat stress might lead to dysbiosis and, in turn, to increased intestinal permeability and metabolic dysfunction [26–28].

The impact of high ambient temperatures on intestinal integrity in chickens has been the focus of several studies in recent years. Heat stress can increase intestinal permeability through a complex process of physiological adaptations [29]. Initially, increasing the peripheral blood flow and reducing intestinal blood supply, heat stress leads to hypoxia, an overproduction of reactive oxygen species (oxidative stress), and cell damage in intestinal tissues [17, 30]. Moreover, the expression of heat shock factors is upregulated, thus leading to an increased production of heat shock proteins, which are crucial for the regulation of protein homeostasis and can be considered a marker of tissue injury [29, 31]. This oxidative stress response is often associated with the disruption and dysfunction of the intercellular junctional complex [29]. Desmosomes, adherens junctions, and tight junctions form the intercellular junctional complex and are hence an important part of the intestinal barrier [32]. Tight junctions are transcellular and paracellular proteins that allow substances to transfer passively across the epithelium following a concentration gradient [33]. Heat stress compromises tight junctions, leading to a condition called “leaky gut”, which in turn facilitates translocation of bacteria from the intestinal tract [15, 34].

Since not only heat stress but also enteric pathogens can disrupt the intestinal barrier [34], it is highly important to investigate the interactions between high ambient temperatures, the caecal microbiota composition, intestinal integrity, and EC infection. Accordingly, this study aimed to understand the impact of EC infection with and without heat stress on gut health during the first 3 weeks of life. Therefore, the objectives of the study were (1) to investigate the course and severity of clinical disease and EC-associated gross lesions, (2) to analyse the development of the caecal microbiota, and (3) to investigate the effects of EC infection and heat stress on intestinal integrity.

## Materials and methods

### Animals and housing

A total of 373 1-day-old Ross 308 broiler chicks (BWE-Brüterei Weser-Ems GmbH & Co. KG, Visbek, Germany) were used in this experiment. Upon arrival, birds were randomly assigned to one of the four study groups and housed under controlled environmental conditions in floor pens at the animal facility of the Clinic for Poultry, University of Veterinary Medicine Hannover, Foundation, Hannover, Germany. Birds had ad libitum access to water and feed. A standard feeding protocol consisting of starter [1–10 days post-hatch (dph)], grower (11–35 dph) and finisher diet (36–42 dph) was applied throughout the trial (Deuka, Deutsche Tiernahrung Cremer GmbH & Co. KG, Duesseldorf, Germany). Birds were exposed to two different ambient temperature profiles. Table 1 shows the thermoneutral and heat stress conditions used throughout the study. The thermoneutral temperature profile was in line with recommendations from Aviagen EPI (Arendonk, Belgium) [35]. The building management system was used to manage and measure temperature settings. At least twice a day, the system was checked for correct functioning. On the day of placement, birds were provided with 24 h of light, and infrared lamps were set up in the pens, which were removed on the third day. Afterwards, the light program was set to 15 h light from 07:30 to 22:30. The Animal Ethics Committee of the University of Veterinary Medicine Hannover, Foundation and the Lower Saxony State Office for Consumer Protection and Food Safety approved the study design (33.19-42505-04-19/3170).

**Table 1:** **Ambient temperature profiles**

Day(s) post-hatch	Thermoneutral conditions (°C) (groups TN and TN + EC)	Heat stress conditions (°C) (groups HS and HS + EC)
1	34	37
3	32	37
5	30	35
8	28	33
11	26	33
14	24	31
17	22	31
20	20	29
23	20	27
26	20	25
29	20	23
32	20	21
35–42	20	20

### Experimental setup

At the day of arrival, the EC-negative status of 10 randomly selected and humanely sacrificed birds was confirmed by bacteriological examination of yolk sac and caecal samples via culture and EC-specific real-time PCR. The remaining 363 birds were randomly divided into four groups, and the EC challenge was performed. The four groups were treated as follows: noninoculated chickens were raised under thermoneutral conditions (TN), noninoculated chickens were raised under heat stress conditions (HS), EC-inoculated chickens were raised under thermoneutral conditions (TN + EC), and EC-inoculated chickens were raised under heat stress conditions (HS + EC; see Table 1). The day-old chicks in the two EC-inoculated groups were inoculated orally with 0.5 mL of an EC suspension (2 × 10^6^ colony-forming units per millilitre (CFU/mL)), whereas the two noninoculated groups received physiological saline orally. Birds were checked at least daily for clinical signs of EC-associated disease, such as apathy, ruffled feathers, and lameness. Animals showing severe signs of apathy or lameness were euthanized, and necropsy was performed (irregular necropsies). Regular necropsies of 20 broilers per group were performed at 7, 14, 21, and 42 dph. The birds were weighed, pathologic lesions were documented, and samples were taken as follows: Amies medium swabs (Hain Lifesciences GmbH, Nehren, Germany) were taken from the heart, liver, and spleen at all necropsies. Additionally, Amies medium swabs were taken from the free thoracic vertebra and the femoral heads after cutting the respective osseous sampling site vertically to expose the bone marrow at 21 and 42 dph. Dry swabs (Applimed SA, Châtel-St-Denis, Switzerland) were taken from the jejunum (2 cm proximal to Meckel’s diverticulum), ileum (2 cm proximal to the ileocecal junction), and caecum (*Corpus caeci*) and stored at −20 °C. At the same intestinal sampling sites, 1 cm of the intestine was taken, digesta was removed, and the sample was placed in 1.5 mL Eppendorf tubes (Sarstedt AG & Co. KG, Nuembrecht, Germany) containing 0.5 mL RNAlater^®^ (Merck KGaA, Darmstadt, Germany) and stored at −80 °C until further use. Caecal samples, including digesta, were taken for caecal microbiota analysis and stored at −20 °C.

### Challenge isolate and preparation of the inoculum

The challenge isolate EC 14/086/4/A was used and prepared for inoculation as previously published [10]. The inoculation dose was set to 2 × 10^6^ colony-forming units per mL (CFU/mL), which was confirmed by determining the total bacterial count.

### EC detection via bacterial isolation and real-time PCR

Microbiological examination was performed as described previously [36]. Briefly, swabs taken from extraintestinal tissues at necropsy were cultured on Columbia colistin-nalidixic acid (CNA) agar (Oxoid GmbH, Wesel, Germany) for 24 h at 37 °C under microaerophilic conditions. Colonies of an EC-typical morphology (small, grey, mucoid colonies with slight alpha-haemolysis) were subcultured and identified as EC via oxidase and catalase testing, Gram staining, and, in case of doubt, by 16S rRNA partial gene sequencing (Microsynth AG, Lindau, Germany [37–39]). DNA was isolated from swabs taken from the jejunum, ileum, and caecum (InnuPrep DNA Mini Kit, Analytik Jena AG, Jena, Germany), and EC-specific real-time PCR was performed exactly as published previously [36].

### Characterization of caecal microbiota

The analysis of the caecal microbiota composition was performed by sequencing the V3-V4 hypervariable region of 16S rRNA genes as described previously [24]. Sequencing results were analysed and classified with RDP Seqmatch using QIIME 2 software [40] with an operational taxonomic unit (OTU) discrimination level set to 97%.

### Electrophysiological measurements

To study the intestinal integrity of the birds in the third week of life, Ussing chamber experiments were performed at 14–18 dph. Each day, four animals (one per group, final sample size *n* = 5 per group at 18 dph) were sacrificed, and the intestines were removed immediately, transferred to ice-cold physiological saline solution to cool down, and then placed in ice-cold carbogen aerated (95% O_2_, 5% CO_2_; pH 7.45–7.47) transport medium (serosal buffer, Additional file 1). Samples from three intestinal segments were prepared immediately after 15 min of transport. Jejunal samples were taken 5 cm proximal to Meckel’s diverticulum, ileal samples were taken 1 cm proximal to the ileocecal junction, and caecal samples were taken at the *corpus caeci.* The respective intestinal segments were placed on an ice-cold glass surface, opened longitudinally, and rinsed with cold physiological saline solution to remove all digesta. Afterwards, the serosal and muscular layers were stripped off, and the mucosa was mounted in the Ussing chamber, exposing an area of 1.00 cm^2^ to the two chamber halves. Each chamber half was filled with 10 mL of a buffer solution at a temperature of 37 °C and a pH of 7.4 under continuous aeration with carbogen to maintain physiological conditions. The composition of the buffer solution differed between the serosal and mucosal sides (Additional file 1). All buffers contained indomethacin (10^–5^ M) to inhibit endogenous prostaglandin production. Each segment was analysed in technical duplicate. The experiments were performed under short-circuited conditions, and the potential differences, tissue conductance (G_t_) and short circuit currents (I_sc_) were continuously recorded by a computer-controlled voltage clamp device (Mussler Scientific Instruments, Aachen, Germany). After a 30-min equilibration period, 10 mM glucose (Merck KGaA, Darmstadt, Germany) and 10 mM mannitol (Sigma Aldrich Inc., St. Louis, MO, USA) were added to the mucosal and serosal sides, respectively, initiating sodium-dependent glucose transport. Moreover, 10^–5^ M carbachol (Sigma Aldrich Inc.) and 5 × 10^–6^ M forskolin (Sigma Aldrich Inc.) were added to the serosal side to initiate chloride secretion with 30 min of recovery after each addition. The experiment was terminated 30 min after the final addition of 10^–4^ M ouabain (Sigma Aldrich Inc.) to the serosal side to test for tissue viability [41].

### mRNA expression of tight junction proteins

mRNA expression of the tight junction proteins claudin-1 (CLDN1), claudin-3 (CLDN3), claudin-5 (CLDN5), claudin-7 (CLDN7), tricellulin (MD2), and zonula occludens-1 (ZO1) in the jejunum and caecum was analysed using an RT‒qPCR assay. RNA was isolated from the intestinal tissue using the Analytik Jena RNA Mini Kit 2.0 (Analytik Jena AG) in accordance with the manufacturer’s instructions, and RNA was stored at −80 °C until further use. Primers and probes were designed exactly as described by von Buchholz et al. [42], and RT‒qPCR was performed with some minor modifications. The Luna^®^ Universal Probe One-Step RT‒qPCR Kit (New England Biolabs GmbH, Frankfurt, Germany) and QuantStudio 3 Real-Time-PCR-System (Thermo Fisher Scientific Inc., Wilmington, NC, USA) were used for RT‒qPCR, and the setup was adapted to the given conditions. Each sample was tested in duplicate for the expression of target genes (CLDN 1, CLDN5, and ZO1 as Multiplex 1; CLDN3, CLDN7, and MD2 as Multiplex 2) and a reference gene (RPL13). Primers and probes for the reference gene were chosen according to Mitra et al. [43]. Additionally, each sample was tested for genomic DNA contamination by running in duplicate without reverse transcriptase for the reference gene. Each run also included two wells with no template control. Ct values were normalized against the reference gene [44].

### Statistical analysis

Statistical analysis of data was performed using the SAS Enterprise Guide (Version 7.15, SAS Institute Inc., Cary, USA), and graphs were created with GraphPad Prism (Version 9.2, GraphPad Software, LLC, San Diego, USA). Descriptive statistics and Fisher’s exact test were used to analyse clinical signs and pathology. EC detection at the different intestinal and extraintestinal sampling sites via culture or real-time PCR was compared between groups using Fisher’s exact test. To determine within-sample diversity (alpha diversity), the diversity estimators Chao1, Shannon, and Simpson index were calculated in QIIME 2 [40]. The alpha diversity indices were further compared between groups using the Kruskal‒Wallis test and Mann‒Whitney U test. Different distance metrics implemented in QIIME 2 were used to visualize beta diversity in principal coordinate analysis (PCoA). The results from electrophysiological measurements (G_t_ and I_sc_) in the Ussing chamber and mRNA expression data of tight junction proteins were compared between groups per intestinal segment using the Kruskal‒Wallis test and the Mann‒Whitney U test. The Benjamini‒Hochberg correction method for multiple testing was used to adjust *p* values where applicable [45], and differences were considered significant at *p* ≤ 0.05.

## Results

### Clinical signs and gross lesions

No clinical signs or gross lesions of EC-associated disease were observed in the two noninoculated groups (Table 2). From 10 dph onwards, nonspecific symptoms, including ruffled feathers and apathy, were observed in 19.1% and 17.2% of chickens in the TN + EC and HS + EC groups, respectively. Lameness was seen less often than apathy in both EC-inoculated groups without any significant difference between them (*p* > 0.05, Table 2). Pericarditis was found significantly more often in the HS + EC group (17.2%) than in the TN + EC group (5.7%, *p* < 0.05). Splenomegaly was found to a similar extent in both EC-inoculated groups (Table 2). Spinal abscesses and osteomyelitis of the femoral heads were found less frequently than lameness in both EC-inoculated groups (Table 2). In some birds, the cause of lameness remained unclear, as examination of additional joints was not performed.

**Table 2:** **EC-associated clinical symptoms and gross lesions**

Group	Clinical signs		Gross lesions				
	Apathy	Lameness	Pericarditis	Perihepatitis	Splenomegaly	Spinal abscess	Osteomyelitis FH
Noninoculated, thermoneutral	1/86 (1.2%)^a^	0/86 (0.0%)^a^	0/86 (0.0%)^a^	0/86 (0.0%)	0/86 (0.0%)^a^	0/40 (0.0%)	0/40 (0.0%)
Noninoculated, heat stress	0/88 (0.0%)^a^	0/88 (0.0%)^a^	0/88 (0.0%)^a^	0/88 (0.0%)	0/88 (0.0%)^a^	0/40 (0.0%)	0/40 (0.0%)
EC-inoculated, thermoneutral	17/89 (19.1%)^b^	6/89 (6.7%)^ab^	5/89 (5.6%)^a^	4/89 (4.5%)	6/89 (6.7%)^ab^	2/40 (5.0%)	0/40 (0.0%)
EC-inoculated, heat stress	15/87 (17.2%)^b^	7/87 (8.1%)^b^	15/87 (17.2%)^b^	2/87 (2.3%)	7/87 (8.1%)^b^	1/42 (2.4%)	2/42 (4.8%)

Statistical analysis was performed per symptom or lesion using Fisher’s exact test and post hoc Benjamini‒Hochberg adjustment for multiple testing (α = 0.05); *p* ≤ 0.05. Different superscript letters indicate significant differences between groups. The analysis included birds examined at regular necropsies and birds euthanized for the Ussing chamber experiments in week three (Days 15–18) as well as birds that died or were euthanized due to animal welfare reasons throughout the experiment. The free thoracic vertebra and the femoral heads (FH) were only examined between 21 and 42 dph, resulting in a smaller sample size for spinal abscesses and osteomyelitis.

In the TN, HS, and TN + EC groups, a number of birds died due to reasons other than EC infection. In the TN group, two birds died due to noninfectious reasons (ascites syndrome and intestinal torsion). In the HS group, four birds died due to noninfectious reasons (ascites syndrome, sudden death syndrome and umbilical hernia). In the TN + EC group, nine birds died due to reasons other than EC infection (weak chick, intestinal invagination, *E. coli* infection, sudden death syndrome and ascites syndrome). However, three birds had to be euthanized due to EC-associated disease (paresis) in the HS + EC group between 21 and 42 dph.

### EC detection in extraintestinal tissues

**Figure 1:**
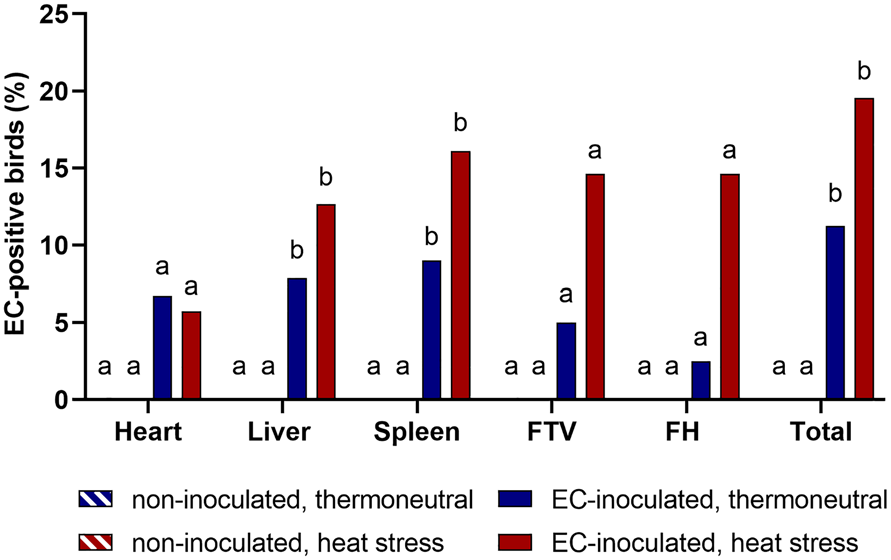
**EC detection on culture in extraintestinal tissues. Groupwise comparisons were performed per tissue by Fisher’s exact test and post hoc Benjamini‒Hochberg adjustment (α = 0.05)**. Different superscript letters indicate significant differences between groups per tissue. *p* ≤ 0.05, *n* = 86 [noninoculated, thermoneutral (TN)], 88 [noninoculated, heat stress (HS)], 89 [EC-inoculated, thermoneutral (TN + EC)], and 87 [EC-inoculated, heat stress (HS + EC)] for heart, liver, spleen, and in total, *n* = 40 (TN, HS, and TN + EC), 42 (HS + EC) for free thoracic vertebra (FTV) and femoral heads (FH). FTV and FH were sampled only at 21 and 42 dph.

In addition to the absence of pathologic lesions, EC was not detected at any extraintestinal sampling site in the two noninoculated groups. In total, 11.2% of the birds in the TN + EC group and 19.5% of the birds in the HS + EC group were EC-positive in either one or more of the examined tissues (Figure 1). The number of EC-positive birds detected from the liver, spleen, free thoracic vertebra, and femoral heads was overall higher in the HS + EC group than in the TN + EC group. However, no significant difference was found between the two EC-inoculated groups for any of the examined tissues (*p* > 0.05). EC was most frequently detected in the spleen, with 8.99% in the TN + EC group and 16.09% in the HS + EC group. Moreover, EC was detected more often than splenomegaly at necropsy (6.7% in the TN + EC group and 8.1% in the HS + EC group). Although pericarditis was seen significantly more often in the HS + EC group (17.2%) than in the TN + EC group (5.6%, *p* < 0.05), EC was isolated to a similar extent from both groups (6.7% from TN + EC and 5.8% from HS + EC). When comparing gross lesions in the FTV and the femoral heads with EC detection rates, more birds were found to be EC-positive than to have gross lesions. At the FTV, 5.0% in the TN + EC group and 2.4% in the HS + EC group had a spinal abscess, whereas 5.0% in the TN + EC group and 14.3% in the HS + EC group were found to be EC-positive at the FTV. Lesions at the femoral heads were found in none of the birds in the TN + EC group and in 4.8% of the birds in the HS + EC group, but slightly more birds were found to be EC-positive on culture in both groups (2.5% in group TN + EC and 14.3% in group HS + EC). The free thoracic vertebra and the femoral heads were only examined between 21 and 42 dph, since EC is not expected to colonize the osseous predilection sites before the fourth week of life. At 21 dph, EC was not detected at the FTV or the femoral heads in the TN + EC group. However, in the HS + EC group, three birds (15%) were EC-positive at the FTV, and four birds (20%) were EC-positive at the femoral heads at 21 dph, but no gross lesions were found. At 42 dph, one (6.3%) and four (20%) birds were EC-positive at the FTV in the TN + EC and HS + EC groups, respectively. A spinal abscess was detected only in the EC-positive bird in the EC + TN group.

### EC detection in the intestine

**Figure 2:**
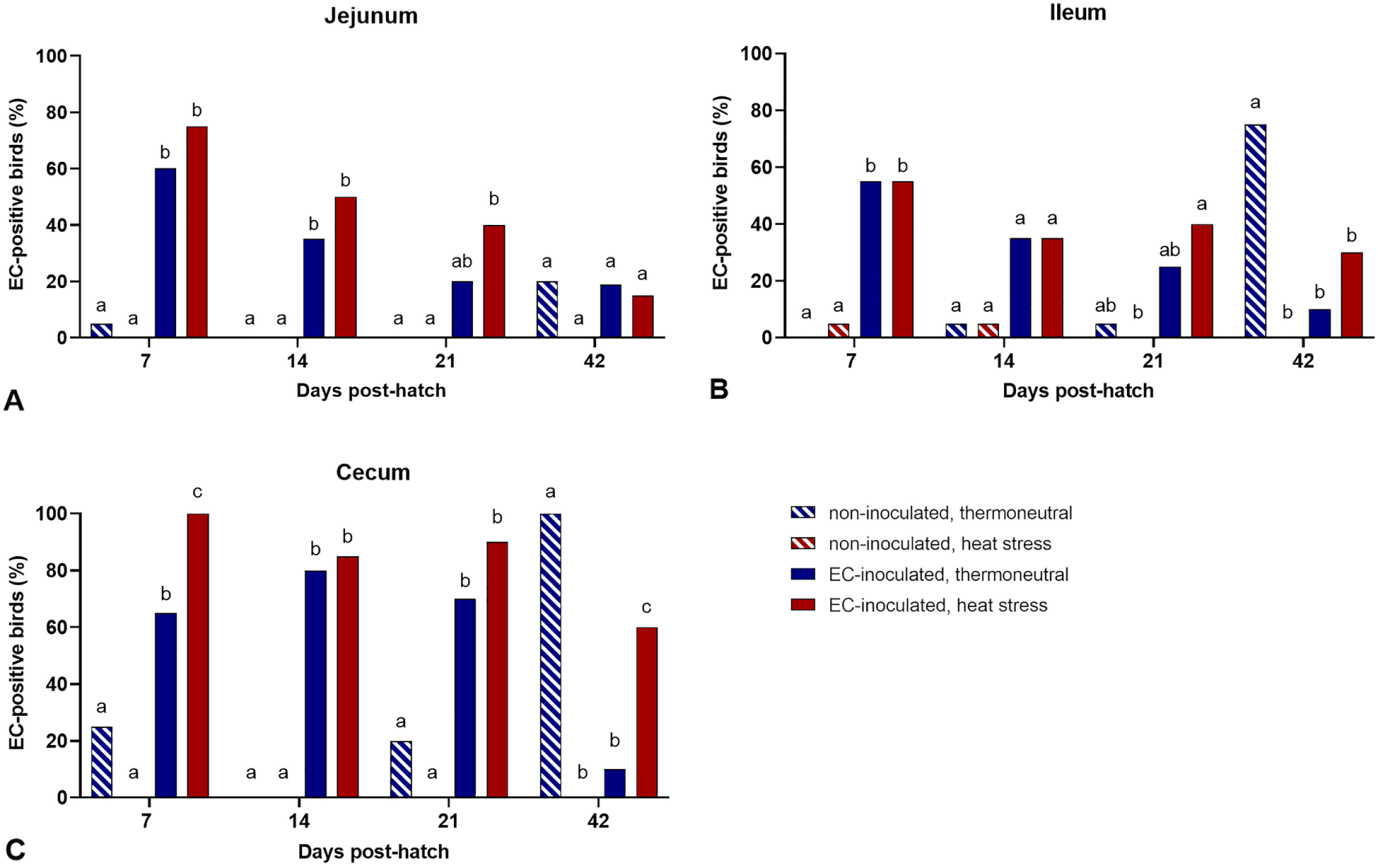
**EC detection along the intestine**. **A** Jejunum, **B** Ileum, **C** Caecum. Groupwise comparisons were performed per tissue by Fisher’s exact test and post hoc Benjamini‒Hochberg adjustment (α = 0.05). Different superscript letters indicate significant differences between groups per sampling day. *p* ≤ 0.05, *n* = 20.

EC was detected via real-time PCR in all three intestinal segments at all sampling days in the TN + EC and HS + EC groups (Figure 2). In general, EC detection rates were highest in the caecum. At 7 dph, 100% of the birds in the HS + EC group were EC-positive in the caecum, which was significantly more than in the TN + EC group, in which 65% of the birds were colonized by EC (*p* < 0.05). At 14 and 21 dph, EC detection rates in the caecum were similar in both groups. At the end of the study, 60% of the birds in the HS + EC group were still EC-positive, whereas only 10% of the birds in the TN + EC group were EC-positive (*p* < 0.05). In the TN group, EC was detected in fewer than 20% of the birds in all three intestinal segments at 7, 14, and 21 dph. Interestingly, all birds in this group were EC-positive in the caecum, 75% in the ileum, and 20% in the jejunum at 42 dph. In the HS group, EC was detected in the ileum of one bird at 7 and 14 dph and not at all at 21 and 42 dph in all three intestinal segments.

### Characterization of the caecal microbiota

A total of 5 869 288 reads were obtained from 153 caecal samples. The mean coverage was 38,361 reads (range 20 030–89 883). These reads were assigned to 1298 operational taxonomic units (OTUs).

#### Alpha diversity and beta diversity

**Figure 3:**
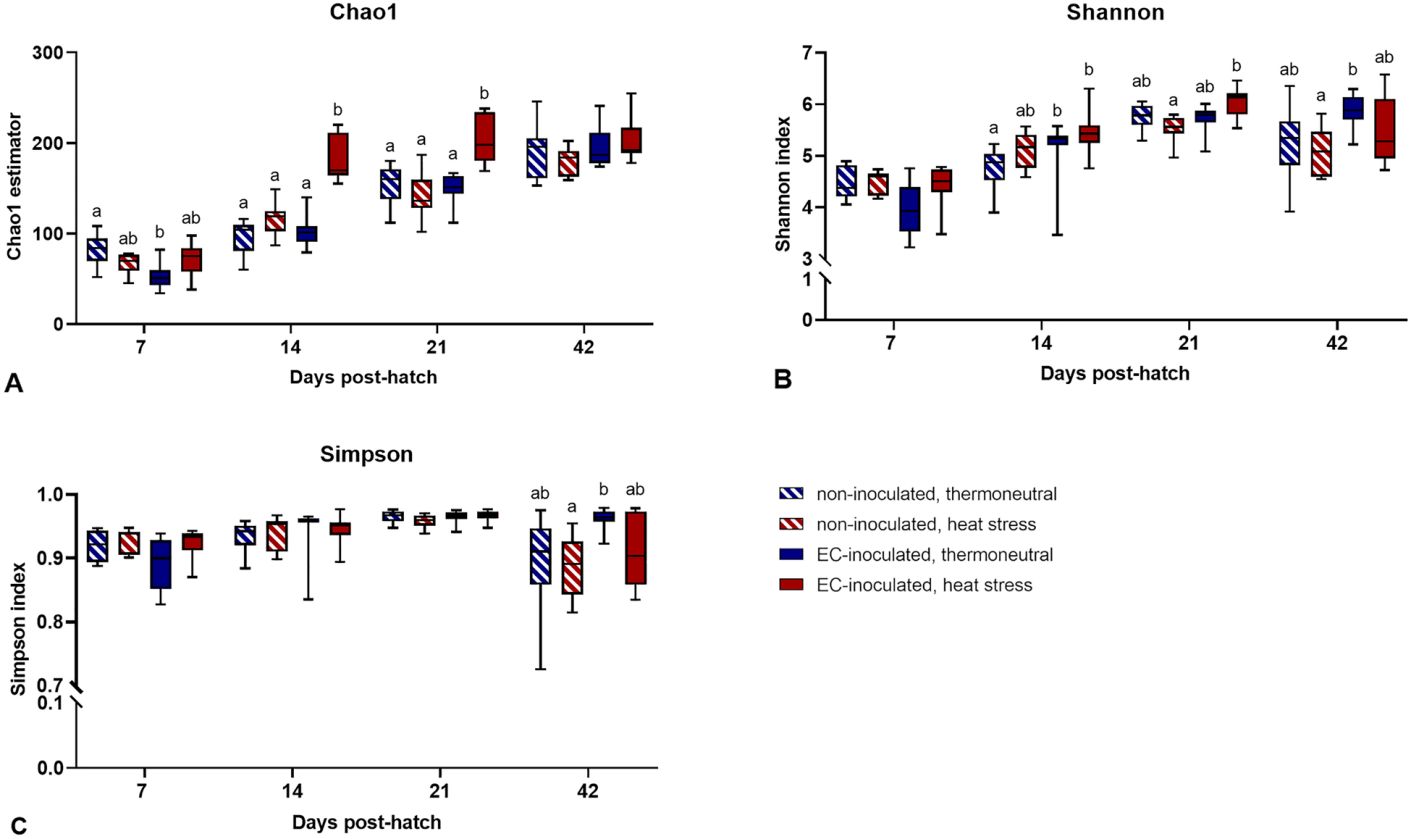
**Alpha diversity estimators**. **A** Chao1 estimator, **B** Shannon index, **C** Gini-Simpson index. Boxes represent the 25^th^ to 75^th^ percentiles, and whiskers extend to the smallest and largest values. Groupwise comparisons were performed per diversity index by the Kruskal‒Wallis test, Mann‒Whitney U test, and post hoc Benjamini‒Hochberg adjustment (α = 0.05). Different superscript letters indicate significant differences between groups per sampling day. *p* ≤ 0.05, *n* = 20.

Regarding the whole trial, there was an increase in species richness (Chao1 estimator) up to 42 dph. At 14 and 21 dph, species richness was significantly higher in the HS + EC group than in the other three groups (*p* < 0.05, Figure 3). At 42 dph, no significant difference in species richness was found between the four groups (*p* > 0.05).

At 7 dph, no significant differences in species diversity (Shannon index) were found (*p* > 0.05, Figure 3). At 14 dph, the caecal microbiota in the two EC-inoculated groups was significantly more diverse than that in the TN group (*p* < 0.05). At 21 dph, species diversity was significantly higher in the HS + EC group than in the HS group (*p* < 0.05). At 42 dph, species diversity was significantly higher in the TN + EC group than in the HS group (*p* < 0.05).

Significant differences in species diversity represented by the Gini-Simpson index were found only at 42 dph, when species diversity was significantly higher in the TN + EC group than in the HS group (*p* < 0.05, Figure 3). At day 42, similar differences were expressed by the Shannon index and the Gini-Simpson index. In summary, species richness differed between the four groups throughout the study period, whereas the species were similarly evenly distributed in all four groups throughout the study period.

In principal coordinate analysis of different distance metrics, no distinct clusters were detected for any group at any time point (data not shown).

#### Relative abundances at the phylum and family levels

Throughout the study, *Firmicutes* was the most abundant phylum in all groups on all study days (>68%). Whereas *Proteobacteria* were most frequently observed at 7 dph (>14%) and 14 dph (>2%) in all groups, *Bacteroidota* became more abundant towards the end of the study (>24%) in all groups except for TN + EC (Additional file 2).

Significant differences in the relative abundances of different phyla were observed mainly at 42 dph (Table 3). The phylum *Firmicutes* was significantly more abundant in the TN + EC group than in the other three groups (*p* < 0.05). At the family level, this difference was mainly due to the higher abundances of the family *Lachnospiraceae* at 42 dph (Table 4). Conversely, the phylum *Bacteroidota* was significantly less abundant in the TN + EC group at 42 dph than in all other groups based on the absence of the family *Rikenellaceae* (*p* < 0.05, Tables 3, 4). The phylum *Verrucomicrobiota* became significantly more abundant in the HS + EC group than in all other groups at 14 and 21 dph (*p* < 0.05).

**Table 3:** **Selected phyla on different sampling days**

Phylum	dph	Noninoculated, thermoneutral (%)	Noninoculated, heat stress (%)	EC-inoculated, thermoneutral (%)	EC-inoculated, heat stress (%)	*p* value
Firmicutes	42	71.8 ± 4.3^a^	68.0 ± 3.0^a^	94.1 ± 2.0^b^	71.2 ± 4.8^a^	0.0004
Proteobacteria	42	2.1 ± 1.0^ab^	0.2 ± 0.1^a^	2.5 ± 1.0^ab^	1.8 ± 0.6^b^	0.0153
Bacteroidota	42	25.8 ± 4.5^a^	30.8 ± 3.0^a^	0.1 ± 0.1^b^	24.5 ± 4.5^a^	0.0001
Verrucomicrobiota	14	0.0 ± 0.0^a^	0.0 ± 0.0^a^	0.0 ± 0.0^a^	8.1 ± 2.1^b^	0.0001
	21	0.0 ± 0.0^a^	0.0 ± 0.0^a^	2.6 ± 0.8^b^	5.8 ± 1.4^b^	0.0001
	42	0.0 ± 0.0^b^	0.6 ± 0.3^a^	2.1 ± 1.2^a^	2.1 ± 1.0^a^	0.0003
Actinobacteriota	7	3.1 ± 1.0^b^	0.1 ± 0.0^a^	0.2 ± 0.1^a^	0.1 ± 0.0^a^	0.0003
	14	0.0 ± 0.0^a^	0.4 ± 0.0^b^	0.3 ± 0.1^bc^	0.3 ± 0.0^c^	0.0001
Desulfobacterota	42	0.0 ± 0.0^a^	0.0 ± 0.0^a^	0.8 ± 0.1^b^	0.1 ± 0.0^c^	0.0001

Kruskal‒Wallis test and Mann‒Whitney U test, post hoc Benjamini‒Hochberg adjustment (α = 0.05). Different superscript letters indicate significant differences between groups per sampling day (*p* ≤ 0.05).

dph: days post-hatch.

**Table 4:** **Selected families on different sampling days**

Family	dph	Noninoculated, thermoneutral (%)	Noninoculated, heat stress (%)	EC-inoculated, thermoneutral (%)	EC-inoculated, heat stress (%)	*p* value
Lachnospiraceae	14	31.4 ± 3.5^a^	30.1 ± 2.3^a^	41.0 ± 4.8^a^	18.9 ± 2.3^b^	0.0032
	21	27.2 ± 1.4^a^	33.4 ± 2.6^ab^	39.9 ± 1.7^b^	25.5 ± 1.4^a^	0.0002
	42	24.2 ± 3.0^ab^	19.9 ± 1.6^a^	33.6 ± 3.1^b^	24.0 ± 2.0^ab^	0.0083
Rikenellaceae	42	25.83 ± 4.52^a^	30.77 ± 2.95^a^	0.02 ± 0.02^b^	24.46 ± 4.51^a^	0.0001
Enterobacteriaceae	42	2.02 ± 0.96^a^	0.20 ± 0.06^b^	2.44 ± 0.94^a^	1.75 ± 0.56^a^	0.0098
Ruminococcaceae	7	7.62 ± 4.86^a^	8.76 ± 2.05^a^	0.67 ± 0.24^b^	7.85 ± 2.99^ab^	0.0125
	14	27.84 ± 3.82^a^	14.50 ± 1.30^b^	13.71 ± 2.55^b^	30.86 ± 2.17^a^	0.0001
	21	27.51 ± 2.53^a^	21.46 ± 3.45^ab^	16.71 ± 1.29^b^	20.17 ± 1.50^ab^	0.0173
Oscillospiraceae	7	10.06 ± 1.98^ab^	10.88 ± 0.99^a^	6.41 ± 0.82^b^	14.71 ± 3.00^ab^	0.0417
	21	22.23 ± 1.50^a^	15.04 ± 1.07^b^	18.57 ± 1.59^ab^	10.66 ± 1.09^c^	0.0001
Lactobacillaceae	14	7.79 ± 1.83^a^	15.73 ± 2.36^b^	9.73 ± 2.39^ab^	11.29 ± 3.10^ab^	0.0354
	21	3.45 ± 1.33^a^	6.45 ± 0.95^b^	1.65 ± 0.35^a^	12.79 ± 1.46^c^	0.0001
Bifidobacteriaceae	7	3.10 ± 0.97^a^	0.00 ± 0.00^b^	0.18 ± 0.14^b^	0.00 ± 0.00^b^	0.0001
Enterococcaceae	14	0.00 ± 0.00^a^	0.14 ± 0.04^ab^	0.07 ± 0.01^b^	0.04 ± 0.02^ab^	0.0032
	21	0.06 ± 0.01^a^	0.01 ± 0.01^b^	0.01 ± 0.01^b^	0.03 ± 0.01^ab^	0.0034
Bacillaceae	21	0.68 ± 0.22^a^	1.71 ± 0.53^ab^	2.03 ± 0.48^b^	0.60 ± 0.19^a^	0.0193

Kruskal‒Wallis test and Mann‒Whitney U test, post hoc Benjamini‒Hochberg adjustment (α = 0.05). Different superscript letters indicate significant differences between groups per sampling day (*p* ≤ 0.05).

dph: days post-hatch.

The most abundant family at 7, 14, and 21 dph was the family *Lachnospiraceae*, followed by *Enterobacteriaceae* (phylum *Proteobacteria*) at 7 dph and *Ruminococcaceae* (phylum *Firmicutes*) at 14 and 21 dph. At 42 dph, *Rikenellaceae* (phylum Bacteroidota) became highly abundant in all groups except for TN + EC. The family *Enterococcaceae* (phylum *Firmicutes*) was less abundant on all sampling days (<1%, Additional file 3 and Table 4).

### Electrophysiological measurements

**Figure 4:**
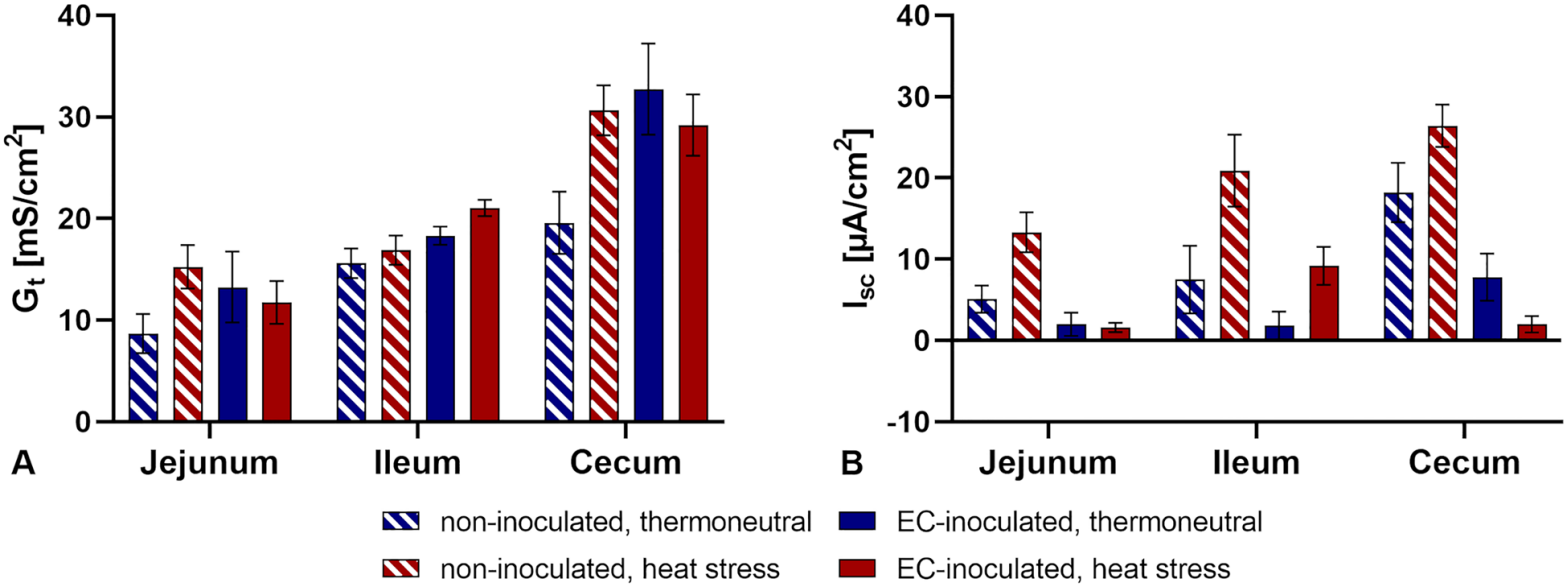
**Basal tissue conductance (G**_**t**_**) and short-circuit currents (I**_**sc**_**) in three intestinal segments**. **A** Tissue conductance (G_t_) in mS/cm^2^. **B** Short-circuit current (I_sc_) in µA/cm^2^. Data are presented as the mean ± standard error of the mean (SEM). No significant differences between groups per intestinal segment were detected by the Mann‒Whitney U test and post hoc Benjamini‒Hochberg adjustment (α = 0.05), *p* ≤ 0.05, *n* = 5.

**Figure 5:**
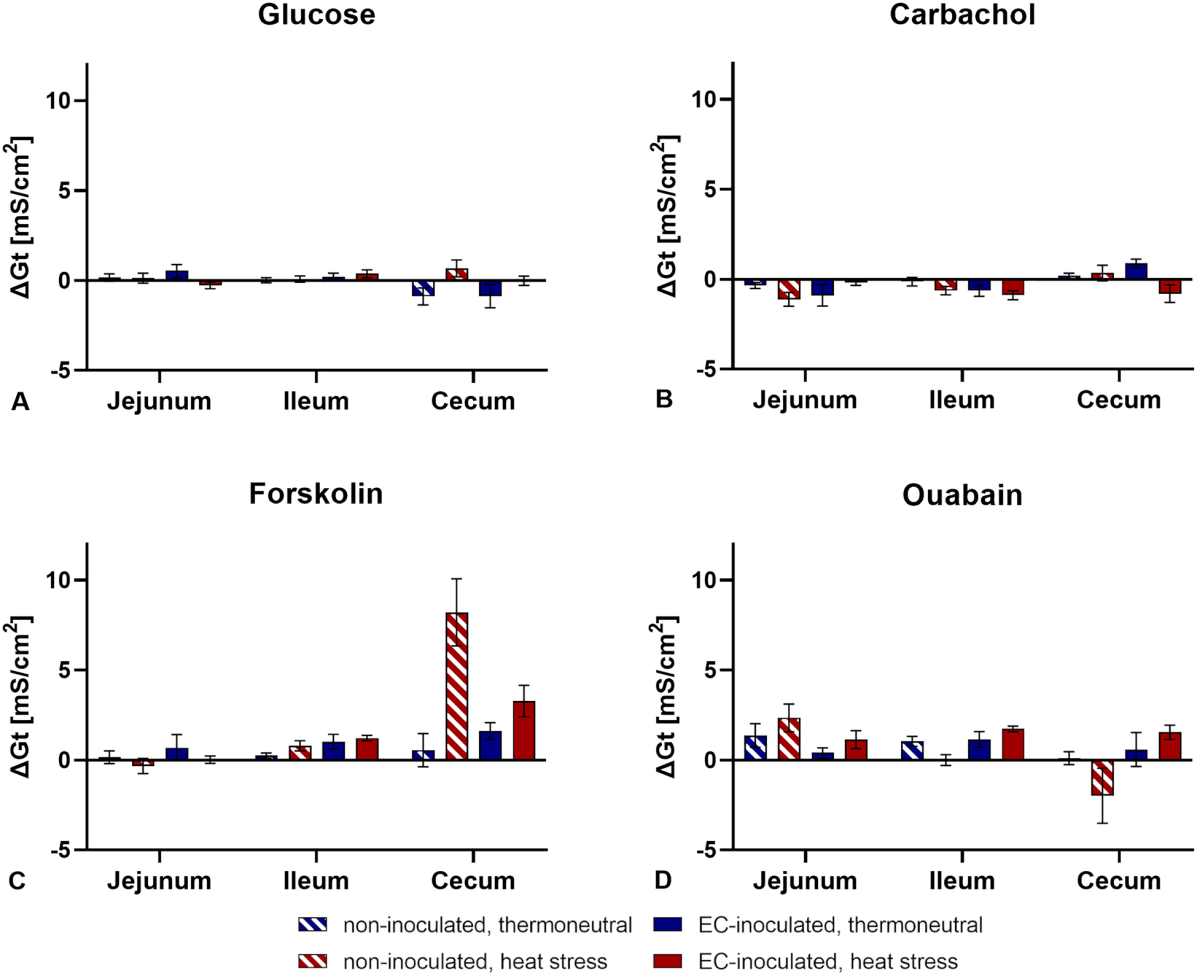
**Maximum changes in tissue conductance (ΔG**_**t**_**) after adding substances**. Substances that were added included **A** glucose (mucosal), **B** carbachol (serosal), **C** forskolin (serosal), and **D** ouabain (serosal). Data are presented as the mean ± standard error of the mean (SEM). ΔG_t_ values did not differ significantly between groups according to the Mann‒Whitney *U* test and post hoc Benjamini‒Hochberg adjustment (α = 0.05), *p* ≤ 0.05, *n* = 5.

**Figure 6:**
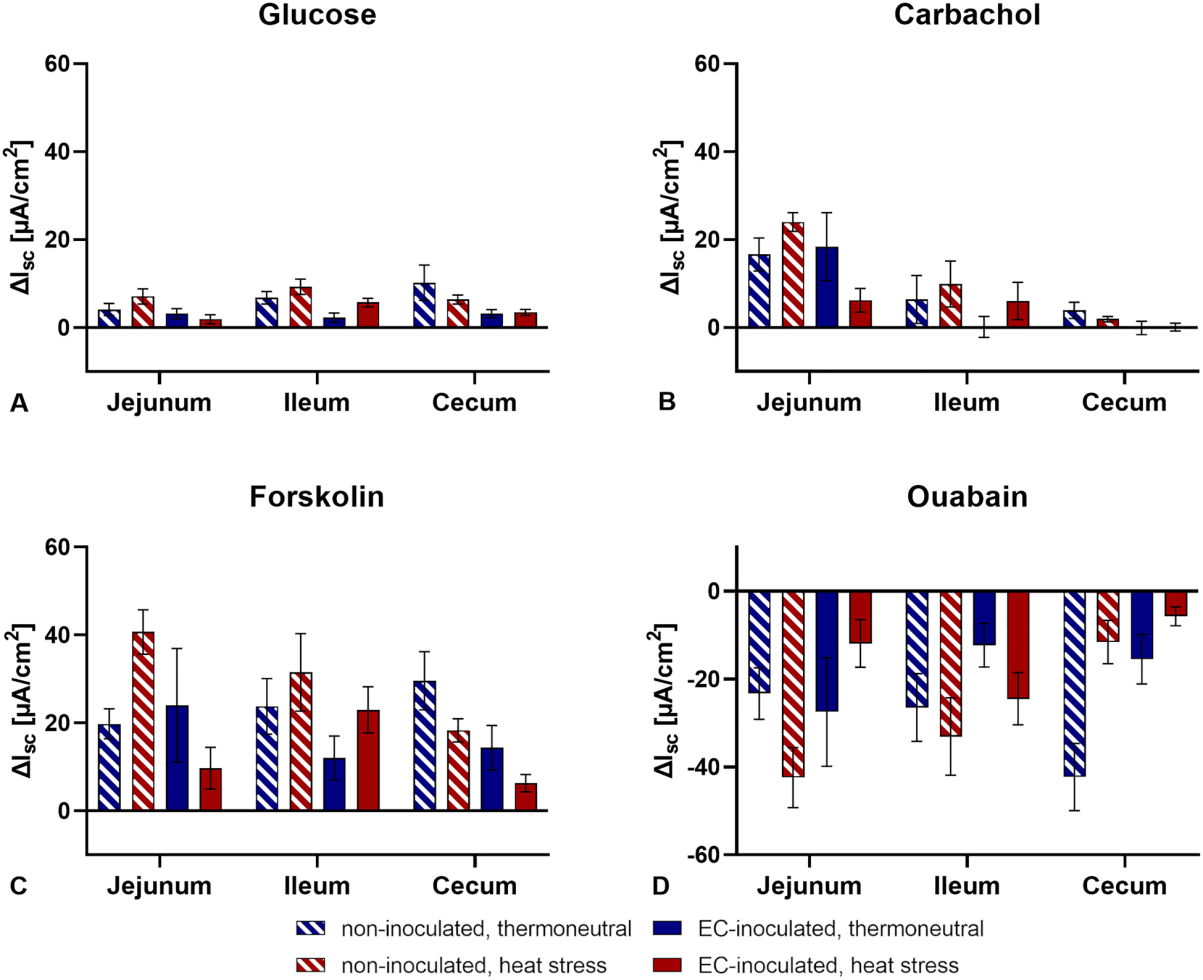
**Maximum changes in short-circuit currents (ΔI**_**sc**_**) after adding substances**. Substances that were added included **A** glucose (mucosal), **B** carbachol (serosal), **C** forskolin (serosal), and **D** ouabain (serosal). Data are presented as the mean ± standard error of the mean (SEM). ΔI_sc_ values did not differ significantly between groups according to the Mann‒Whitney U test and post hoc Benjamini‒Hochberg adjustment (α = 0.05), *p* ≤ 0.05, *n* = 5.

Basal tissue conductance (G_t_) and basal short-circuit currents (I_sc_) were measured continuously during the experiment. The lowest basal G_t_ and I_sc_ values were observed in the jejunum, and the highest values were observed in the caecum (Figure 4). No significant differences in basal G_t_ or I_sc_ values between groups were found for each intestinal segment (*p* > 0.05). Adding glucose (mucosal), carbachol (serosal), forskolin (serosal), and ouabain (serosal) did not alter G_t_ significantly in any of the groups (*p* > 0.05, Figure 5). Additionally, no significant differences in ΔG_t_ between the groups in any intestinal segment were found after adding the abovementioned substances (*p* > 0.05). Although changes in I_sc_ values were observed after adding the substances, these were not statistically significant (*p* > 0.05, Figure 6). Furthermore, no significant differences in ΔI_sc_ were found between groups (*p* > 0.05).

### mRNA expression of tight junction proteins in different intestinal sections

**Figure 7:**
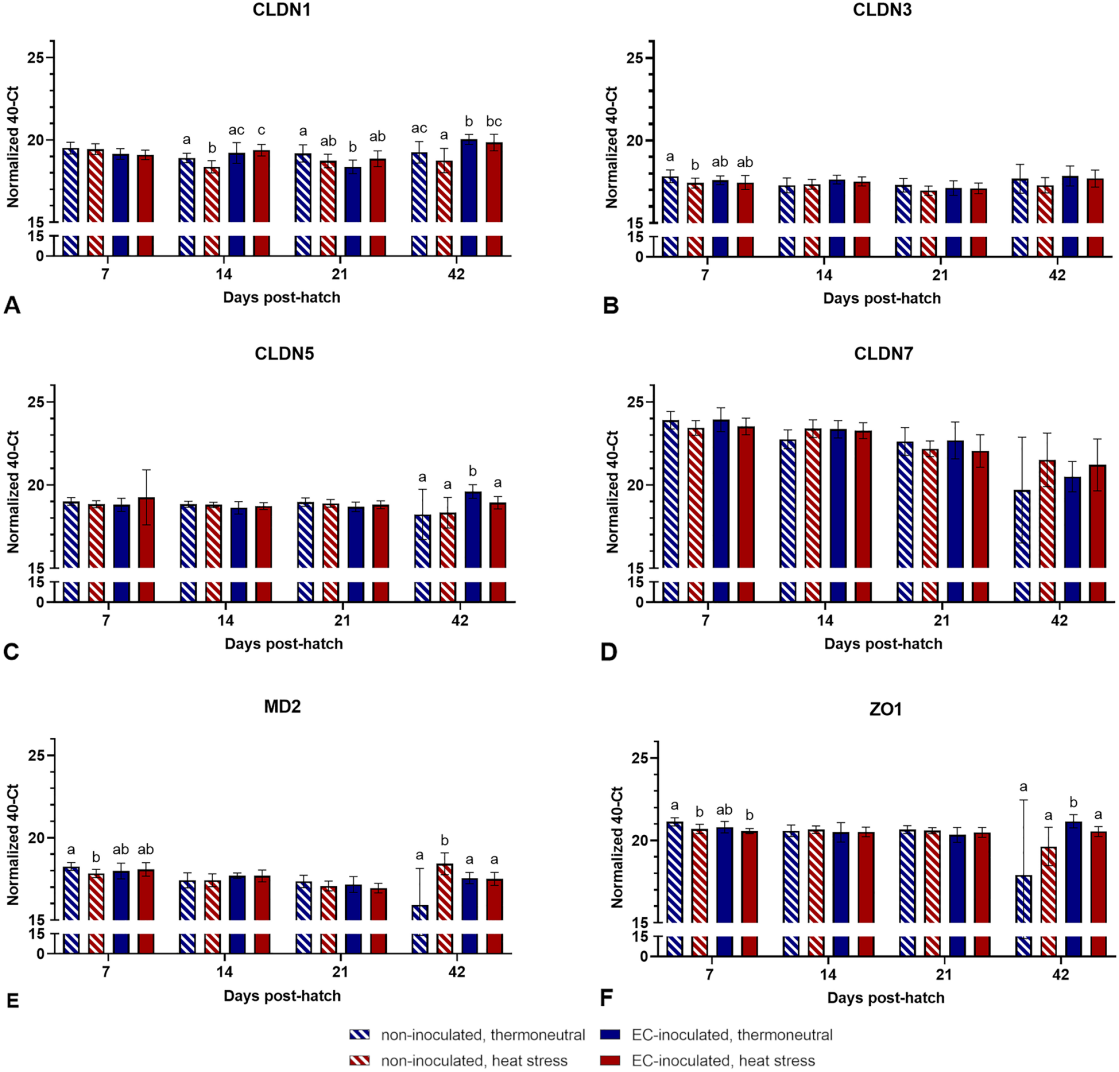
**mRNA expression levels of different tight junction proteins in the jejunum**. Data are presented as the mean ± standard deviation. Groupwise comparisons were performed per tight junction protein by the Kruskal‒Wallis test, Mann‒Whitney U test, and post hoc Benjamini‒Hochberg adjustment (α = 0.05). Different superscript letters indicate significant differences between groups per sampling day. *p* ≤ 0.05, *n* = 20.

No significant differences in jejunal tight junction protein mRNA expression levels between the different groups were observed at most time points (*p* > 0.05, Figure 7). However, at 7 dph, the mRNA encoding CLDN3, MD2, and ZO1 was expressed at significantly higher levels in the TN group than in the HS (CLDN3, MD2, and ZO1) and HS + EC (ZO1; *p* < 0.05) groups. At 42 dph, the mRNA expression levels of CLDN1, CLDN5, and ZO1 were significantly higher in the TN + EC group than in the noninoculated groups (CLDN1, CLDN5, and ZO1) and the HS + EC group (CLDN5 and ZO1; *p* < 0.05). Nonetheless, the mRNA expression of MD2 was significantly higher in the HS group than in all other groups at the same time point (*p* < 0.05).

**Figure 8:**
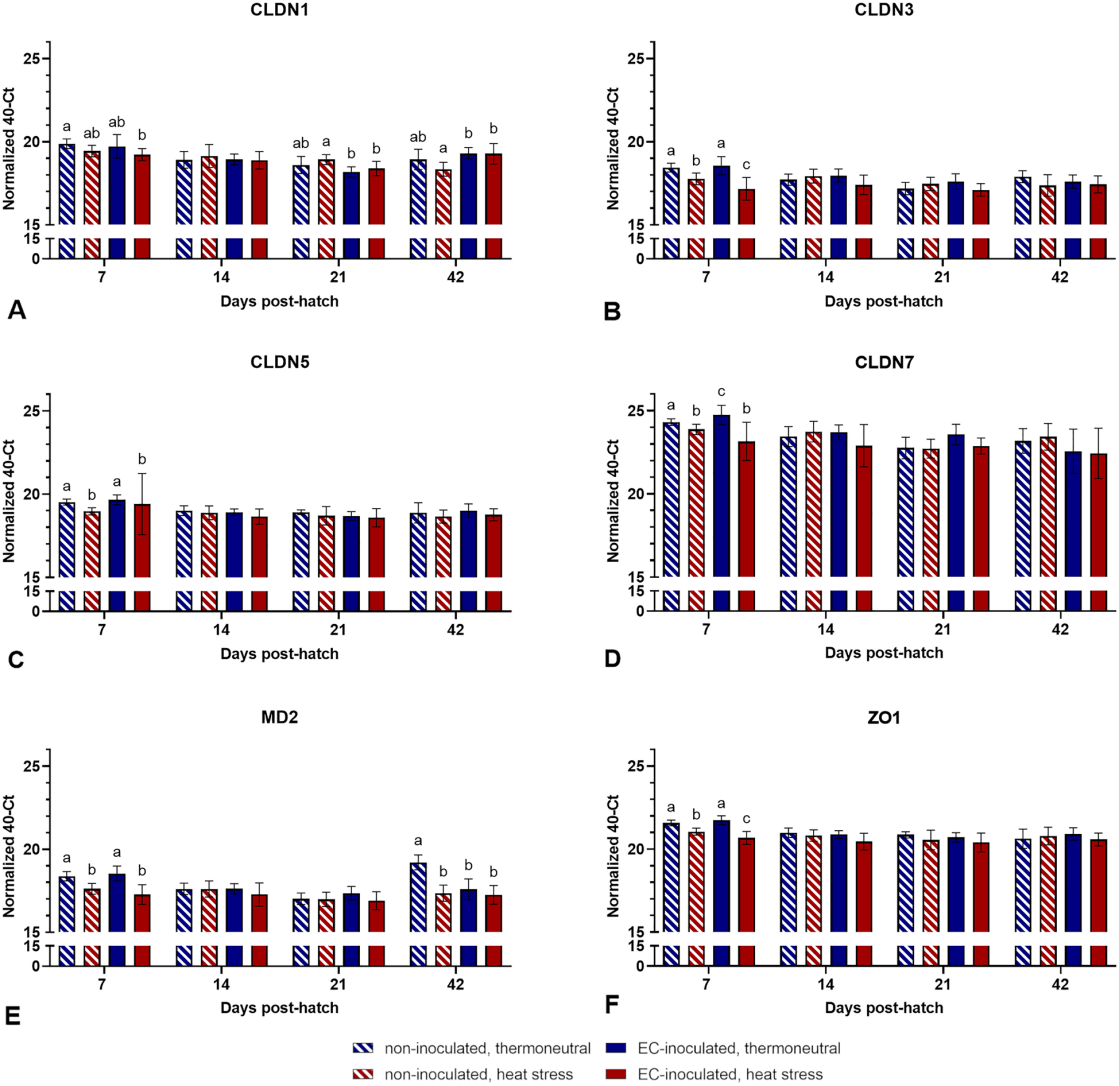
**mRNA expression levels of different tight junction proteins in the caecum**. Data are presented as the mean ± standard deviation. Groupwise comparisons were performed per tight junction protein by the Kruskal‒Wallis test, Mann‒Whitney U test, and post hoc Benjamini‒Hochberg adjustment (α = 0.05). Different superscript letters indicate significant differences between groups per sampling day. *p* ≤ 0.05, *n* = 20.

A clear pattern of significant differences in tight junction mRNA expression was observed in the caecum (Figure 8). At 7 dph, significantly higher normalized 40-Ct values were observed in the thermoneutral groups than in the heat-stressed groups for all six investigated tight junction proteins (*p* < 0.05). Further differences were observed for CLDN1 expression levels at 21 and 42 dph and for MD2 at 42 dph (*p* < 0.05; Figure 8). While CLDN1 expression was higher in the HS group than in both EC-inoculated groups at 21 dph (*p* < 0.05), it was significantly lower at 42 dph when comparing the HS group to the TN + EC and HS + EC groups (*p* > 0.05). MD2 expression was significantly higher in the TN group than in all other groups at 42 dph (*p* < 0.05).

## Discussion

Despite the economic importance of EC infection in broiler production, EC pathogenesis is not fully understood. To date, the role of the caecal microbiota, intestinal integrity, and predisposing factors such as heat stress during EC pathogenesis remain largely unknown. This study aimed to understand the interaction between EC infection and gut health with and without underlying heat stress conditions.

### Heat stress conditions enhance and accelerate EC-associated disease

The onset of clinical signs was observed at 10 dph in both EC-inoculated groups, but more severe clinical signs were found in the HS + EC group, leading to euthanasia of three of 87 birds prior to the last sampling day. Additionally, EC was detected at the osseous predilection sites earlier in the HS + EC group than in the TN + EC group. Although significant differences in EC detection rates via culture were lacking, the percentage of EC-positive birds was higher in the HS + EC group (19.5%) than in the TN + EC group (11.2%) in total and in all extraintestinal organs except for the heart. Accordingly, heat stress might not only slightly enhance the number of affected birds but also accelerate and exacerbate the course of the disease in these animals. The nonspecific nature of the EC-associated disease and our study design focusing on the early infection phase complicate the interpretation of the results to some extent. EC detection rates in both inoculated groups were lower than in previous studies where more than 23% of the birds were EC-positive in either one or more of the examined extraintestinal tissues (heart, liver, spleen, FTV, femoral heads) [10, 36]. One of the reasons for this discrepancy may be the termination of the experiment for 75% of the birds before 21 dph, leaving only 20 birds per group that could fully develop chronic lesions of the EC-associated disease. EC was isolated from osseous predilection sites in some birds in this study, although no macroscopic lesions were detected. It may be suggested that emerging lesions could have been detected by histological examination, since previous studies reported that EC can be detected before gross lesions develop [10, 36]. Consequently, this may explain the differences observed between clinical signs, gross lesions, and EC detection rates in the present study.

Intestinal EC detection rates were highest in the caecum, which is in accordance with previously reported EC colonization patterns [46]. Heat stress may be the reason for the higher caecal EC detection rates in the HS + EC group at 7 dph. Furthermore, the lower caecal EC detection rate in the TN + EC group may have reduced the chance of translocation. This might be an explanation for the lower rates of clinical signs, gross lesions, and EC detection in extraintestinal tissues during the septic phase. Intestinal colonization is thought to be a crucial step in EC pathogenesis [11]. It may be assumed that heat stress deteriorated colonization resistance against the pathogenic EC strain and promoted translocation to the bloodstream, resulting in an increased incidence of the disease.

The effect of heat stress on other bacterial diseases of chickens has been the focus of several studies in recent years. Tsiouris et al*.* [14] found that heat stress might be a predisposing factor for necrotic enteritis. They observed a relative, but not significant, trend towards more severe lesions and an increased incidence of necrotic enteritis due to cyclic acute heat stress. The lack of significant effects was assumed to be caused by the use of a cyclic heat stress protocol, which might have provided recovery periods for the birds [14]. In contrast, a study conducted several years prior to that study indicated no impact of heat stress on necrotic enteritis in broilers [47]. However, since infection and heat stress protocols differed between the studies, they are difficult to compare [14]. In *E. coli* infection, heat stress has been shown to increase the inflammatory response in comparison to thermoneutral conditions [16]. Conversely, another study indicated that cyclic acute heat stress affected the immune system but not resistance against *E. coli* infection [48]. Cyclic acute heat stress may also promote translocation of *Salmonella* spp. and endotoxins due to increased intestinal permeability [15, 49]. Based on these different studies and the present data, it can be concluded that heat stress is a predisposing factor for enteric pathogens, including EC.

Heat stress may adversely affect immunity, productivity, and animal welfare [50, 51]. Existing studies on heat stress effects in broilers mostly focus on performance during the second half of the production cycle, since average daily weight gain is highest and effects are most prominent during that period [52–55]. Accordingly, the comparability of the present study with other studies is limited. The first 3 weeks of life are supposed to be crucial in EC pathogenesis [11], but even the egg incubation period could be important for disease resistance against EC. Heat stress during the egg incubation period may negatively affect bone development [56]. However, increased egg incubation temperature around hatching does not affect the incidence of OCD in the FTV, which is considered to be another predisposing factor for EC-associated disease [57].

Stress and immunosuppression have previously been shown to be influencing factors during pathogenesis of the so-called bacterial chondronecrosis and osteomyelitis (BCO) in broilers [58]. Among other bacteria, EC is considered a causative agent of BCO [59]. In the respective study, repeated dexamethasone injections were used to mimic immunosuppression, and the impact of repeated episodes of heat stress on BCO lesions was investigated. While the present investigation exposed birds to chronic heat stress in the first 3 weeks of life, Wideman et al. used a different model consisting of 3 days of heat stress per week from 21 to 42 dph [58]. This type of heat stress accelerated and exacerbated BCO lesions at the femoral and tibial heads at least within the fourth and fifth weeks of life. Afterwards, no differences could be observed, suggesting acclimation to heat stress conditions [58]. These adaptation processes might also explain the lack of significant effects of permanent heat stress in the present study. In a study investigating adaptation to repeated episodes of heat stress, it was shown that acclimation is a key factor in managing and surviving subsequent acute heat stress phases [60].

Finally, EC is thought to be an opportunistic pathogen with several factors predisposing broilers to EC-associated disease [61]. Heat stress alone does not significantly increase the incidence of the associated disease but may still contribute to EC pathogenesis in the field.

### Commensal EC strains from an unknown source tend to colonize noninoculated birds

Interestingly, the TN group was completely colonized by EC at the end of the study. It may be suggested that birds in this group were colonized by a commensal EC strain from an unknown source. Although the experiment was performed under strict hygiene conditions, it is possible that this strain was introduced into the isolation unit via biotic or abiotic factors. Similar observations were made in two previous studies, but we still lack identification of the respective strain [10, 36]. Isolation of EC strains from caecal contents can be challenging due to overgrowth by other bacteria, such as enterococci, streptococci, and *E. coli* [62]. There is no selective medium for EC available, and although highly specific, our real-time PCR assay does not allow us to distinguish different EC strains [46, 63]. To better understand the colonization patterns of different EC strains, culture-independent methods that allow differentiation of EC strains should be implemented in future studies. This approach might further help to distinguish commensal and pathogenic strains that can appear within experiments even under controlled environmental conditions [10].

### EC infection and heat stress have a limited effect on caecal microbiota

The caecum harbours the most complex and diverse microbiota within the intestinal tract of chickens due to the availability of nutrients such as polysaccharides and longer transit times in this compartment. Interactions within the microbiota and between the microbiota and the host are highly complex and may be influenced by various factors [18, 19]. Accordingly, the differences in alpha diversity as well as caecal composition at the phylum and family levels may be explained by chance rather than by EC infection and temperature conditions. During the initial infection phase at 7 dph, species diversity was not altered by heat stress or EC infection. Minor differences in species richness and relative abundances at the phylum and family levels were mainly observed between the two thermoneutral groups, indicating a possible effect of EC infection at that time point. Differences at later time points, such as higher richness at 14 and 21 dph in the HS + EC group than in the other groups, might be associated with heat stress, since it has been reported that species richness is higher in birds hatched during the summer months [25]. However, there is also an increased risk of pathogenic and commensal bacteria being introduced by insects during summer, as reported for *Campylobacter* spp. [64]. If heat stress was the only factor influencing species richness, a similarly high species richness as observed in the HS + EC group would have been expected for the HS group. Moreover, lower species diversity was observed in the HS group than in the TN + EC group at 42 dph. Since similar temperature conditions in all four groups were reached a week earlier at 35 dph, it is unlikely that this difference was caused by heat stress. Instead, other factors may have influenced species diversity at that time point, especially since birds were housed in separate isolation units. Although housing conditions, except for temperature, were similar in each group, different development of caecal microbiota is possible. It has been described previously that the caecal microbiota composition may be affected by various factors and varies greatly even between highly comparable experimental setups [19]. In recent studies, enterococci were shown to form a minority subpopulation within the caecal microbiota in the first weeks of life [36, 65]. Furthermore, it was reported that EC can induce disease in experimentally infected chickens despite its low abundance in the caecum [36]. This is in accordance with the findings of the present study, which suggest that EC infection had no direct impact on the caecal microbial composition. In addition, the genus *Enterococcus* generally seems to be less influenced by interventions such as antibiotic treatment [36], administration of probiotic *Bacillus* strains, palm oil, and water soluble esterified butyrins [65], or synbiotic administration under heat stress conditions [66]. Based on our observations, heat stress also elicits no clear effects on the family *Enterococcaceae*. Finally, in our study, there was no clear evidence that either EC infection or ambient temperature conditions had a decisive impact on the caecal microbiota.

### Heat stress and EC infection may impair intestinal integrity

To investigate the influence of heat stress and EC infection on intestinal integrity, Ussing chamber experiments were performed in the third week of this study. Only slight changes in tissue conductance without significant differences between groups were observed, which implies that intestinal permeability was not altered by heat stress or enterococcal infection in the third week of life. Although the added substances triggered their receptors and the associated ion transport mechanisms, the observed changes in the I_sc_ values were not significant compared to the basal values, and no significant differences between groups were detected. An influence of heat stress or enterococcal infection on intestinal integrity and active transport mechanisms in the third week post-hatch may thus be ruled out. However, data obtained in these Ussing chamber experiments are limited due to the small sample size (*n* = 5) and the restriction of the time point to the third week of the study. Small sample sizes in Ussing chamber experiments generally limit the outcome and interpretation of results [67]. Since heat stress was applied in the first week of the trial and an adaptation of the birds to the high ambient temperatures cannot be excluded, the third week as a time point for the Ussing chamber experiment may simply be too late. Additionally, EC is expected to escape the intestine soon after infection in the first to second week of life [11]. Earlier investigations of intestinal integrity via Ussing chamber experiments were considered but not performed because of the small size of the intestine. For that reason, the mRNA expression of tight junction proteins was investigated at all sampling days, and a greater sample size was chosen.

Significant differences indicating an effect of heat stress and EC infection on the mRNA expression of tight junction proteins were observed mainly in the caecum at 7 dph. All six investigated tight junction proteins were expressed at significantly lower levels in the heat-stressed groups than in the thermoneutral groups. Heat stress has been shown to influence tight junction integrity in several studies in modern broiler lines [55, 68, 69]. However, adaptation to heat stress exposure is reached within 7 days, which may explain the absence of similar significant differences in tight junction mRNA expression after the first sampling day at 7 dph [60]. Interestingly, as discussed above, EC was shown to be mainly present in the caecum, with higher detection rates observed in the HS + EC group than in the TN + EC group at 7 dph. In relation to mRNA expression data, it may be suggested that the caecum is not only the most favourable location of colonization but also the location of translocation to the bloodstream, which is one of the crucial steps in EC pathogenesis [11]. Intestinal integrity impairment by heat stress may promote this translocation.

Differences observed for expression levels of CLDN1 at 14 and 21 dph and CLDN1, CLDN5, MD2, and ZO1 at 42 dph in the jejunum and caecum may only partly be explained by EC infection. An effect of heat stress at these time points seems to be unlikely. Similar to the caecal microbiota, tight junction protein expression may be influenced by several factors in addition to heat stress and infection. Delayed feeding post-hatch [70], probiotic supplementation [71, 72], and different dietary compounds [73, 74] have been shown to influence tight junction mRNA expression. Moreover, it was shown in previous studies that EC pathogenesis is not necessarily associated with histologically detectable intestinal damage [9, 11]. Thus, it is still possible that EC does not affect intestinal integrity at all. Finally, based on the results discussed here, further experiments on EC pathogenesis should focus on the first 2 weeks post-infection to identify possible effects of EC on intestinal integrity and translocation mechanisms of EC.

In conclusion, heat stress seemed to accelerate EC translocation and make birds susceptible to more severe disease development in the present study. Heat stress may predispose broilers to EC-associated disease in interaction with other, as yet unknown, environmental and host factors. No clear effects of heat stress and EC infection on the caecal microbiota were found in this study. Nevertheless, EC may be assumed to be a key species in affected birds despite being less abundant in the caecal microbiota. The first 2 weeks of life seem to be the critical time period for EC translocation to the bloodstream. Disruptive effects of heat stress on tight junction integrity in the first week of life may have contributed to increased extraintestinal EC detection rates in the present study. Our research contributes to understanding the unknown role of intestinal integrity and caecal microbiota in EC infection. Possible interactions of EC with the intestinal barrier should be further investigated to better understand the pathogenesis of EC-associated disease.

## Supplementary Information

**Additional file 1.** **Composition of serosal and mucosal buffer solutions used for the Using chamber experiments**.

**Additional file 2.** **Relative abundance (%) of caecal microbiota at the phylum level**.

**Additional file 3.** **Relative abundance (%) of caecal microbiota at the family level.**

## Data Availability

All data generated or analysed during this study are included in this published article and its additional files.
