# Supplementary material for: Influence of heat stress on intestinal integrity and the caecal microbiota during Enterococcus cecorum infection in broilers
Source: Vet Res. 2022 Dec 16;53:110. doi: 10.1186/s13567-022-01132-y (PMC9756510; doi:10.1186/s13567-022-01132-y)
Supplement: Supplementary file 1 — Additional file 1. Composition of serosal and mucosal buffer solutions used for the Using chamber experiments. [file 13567_2022_1132_MOESM1_ESM.pdf]

Additional file 1: Composition of serosal and mucosal buffer solutions used for the Ussing chamber experiments.

Buffer ingredients	Serosal buffer [mmol/L] (all gut sections)	Mucosal buffer [mmol/L] (jejunum)	Mucosal buffer [mmol/L] (ileum and cecum)
NaCl	113.6	113.6	53.6
KCl	5.4	5.4	5.4
HCl	0.2	0.2	0.2
$MgCl_2 \times 6\ H_2O$	1.2	1.2	1.2
$CaCl_2 \times 2\ H_2O$	1.2	1.2	1.2
$NaHCO_3$	21.0	21.0	21.0
$Na_2HPO_4 \times 2\ H_2O$	1.5	1.5	1.5
Glucose	10.0	-	-
Mannitol	2.0	2.0	2.0
HEPES[1]	7.0	20.0	10.0
Sodium gluconate	6.0	-	6.0
NaOH	-	6.0	-
Sodium acetate	-	-	36.0
Sodium propionate	-	-	15.0
Sodium butyrate	-	-	9.0

[1](*N*-2-hydroxyethylpiperazine-*N*’-2-ethansulfonic acid)
