# Supplementary figures and images for: Influence of heat stress on intestinal integrity and the caecal microbiota during Enterococcus cecorum infection in broilers
Source: Vet Res. 2022 Dec 16;53:110. doi: 10.1186/s13567-022-01132-y (PMC9756510; doi:10.1186/s13567-022-01132-y)

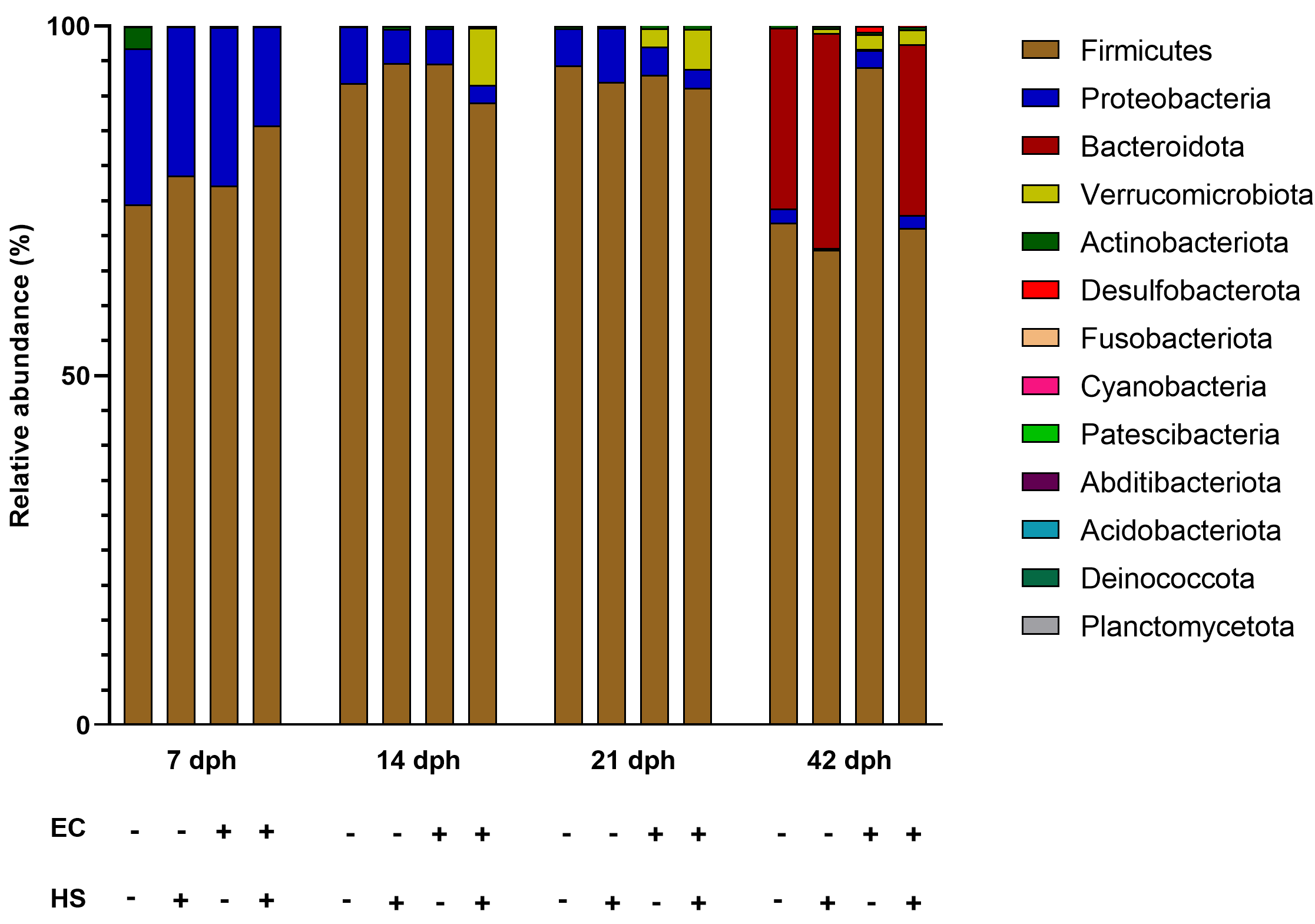

Supplement: Supplementary file 2 — Additional file 2. Relative abundance (%) of caecal microbiota at the phylum level. [file 13567_2022_1132_MOESM2_ESM.tif]

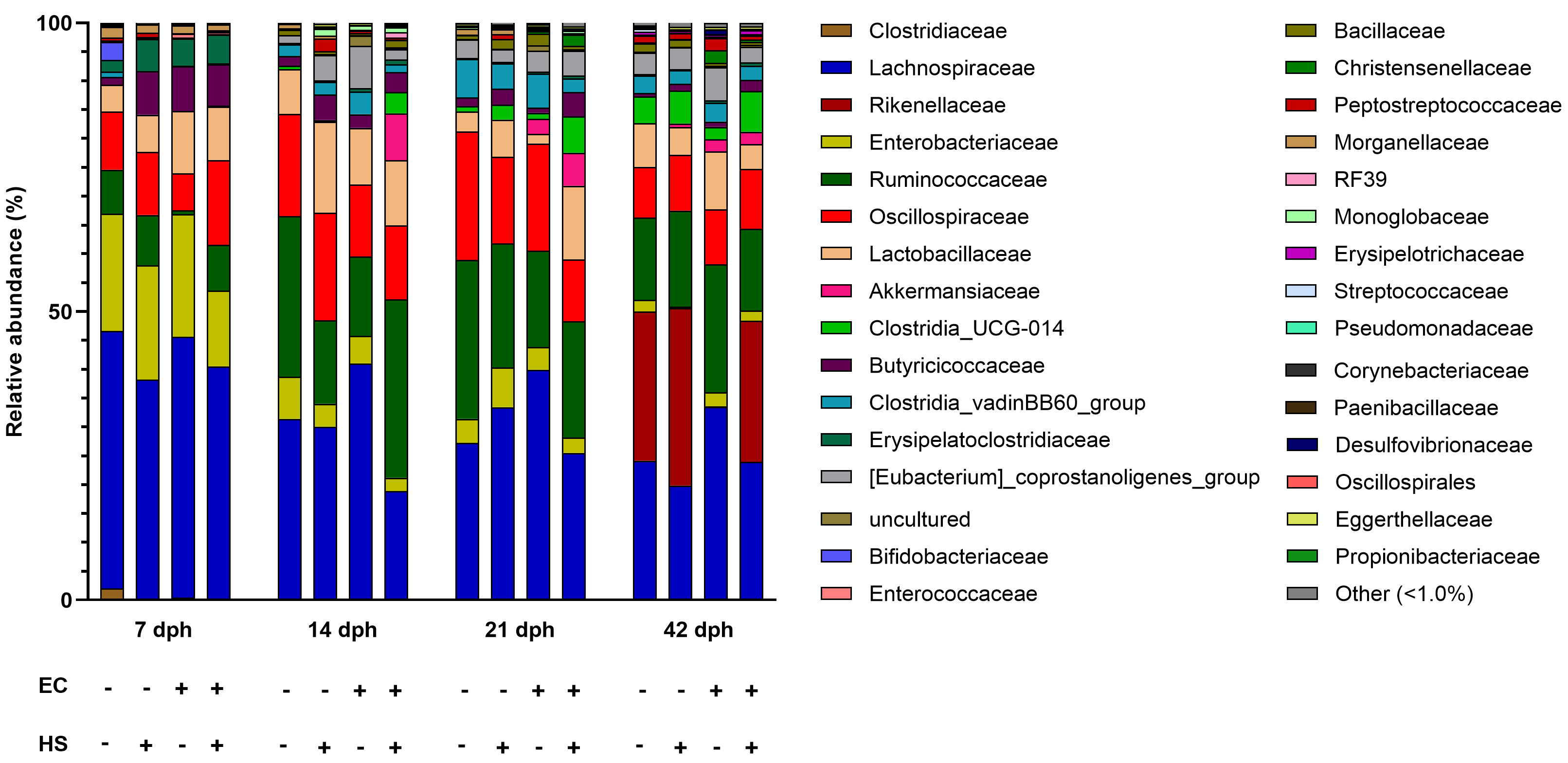

Supplement: Supplementary file 3 — Additional file 3. Relative abundance (%) of caecal microbiota at the family level. [file 13567_2022_1132_MOESM3_ESM.tif]
